# Genetic Polymorphisms in CYP2C19 Cause Changes in Plasma Levels and Adverse Reactions to Anlotinib in Chinese Patients With Lung Cancer

**DOI:** 10.3389/fphar.2022.918219

**Published:** 2022-06-22

**Authors:** Tingfei Tan, Gongwei Han, Ziwei Cheng, Jiemei Jiang, Li Zhang, Zitong Xia, Xinmeng Wang, Quan Xia

**Affiliations:** ^1^ Department of Pharmacy, The First Affiliated Hospital of Anhui Medical University, Hefei, China; ^2^ School of Pharmacy, Anhui Medical University, Hefei, China; ^3^ School of Pharmacy, Anhui University of Chinese Medicine, Hefei, China; ^4^ Department of Pharmacy, The Second Affiliated Hospital of Bengbu Medical College, Bengbu, China

**Keywords:** anlotinib, lung cancer, CYP450 gene polymorphisms, plasma concentration, adverse reactions

## Abstract

**Background:** Anlotinib is a small molecular multi-targeting tyrosine kinase inhibitor. Growing evidence indicates that treatment efficacy, and toxicity varies considerably between individuals. Therefore, this study aimed to investigate the relationship between cytochrome P450 (CYP450) gene polymorphisms, drug concentrations, and their adverse reactions in anlotinib-treated patients with lung cancer.

**Methods:** We enrolled 139 patients with lung cancer, treated with anlotinib. Twenty loci in the following five genes of the CYP450 family were genotyped: CYP450 family 3 subfamily A member 5 (CYP3A5), 3 subfamily A member 4 (CYP3A4), 2 subfamily C member 9 (CYP2C9), 2 subfamily C member 19 (CYP2C19), and 1 subfamily A member 2 (CYP1A2). Data on adverse reactions were collected from patients, and plasma anlotinib concentrations were measured.

**Results:** There were significant variances in plasma trough concentration (3.95–52.88 ng/ml) and peak plasma concentration (11.53–42.8 ng/ml) following administration of 8 mg anlotinib. Additionally, there were significant differences in the plasma trough concentration (5.65–81.89 ng/ml) and peak plasma concentration (18.01–107.18 ng/ml) following administration of 12 mg anlotinib. Furthermore, for CYP2C19-rs3814637, the peak plasma concentrations of mutant allele T carriers (TT+CT) were significantly higher than those of wildtypes (CC). For CYP2C19-rs11568732, the peak plasma concentrations of the mutant allele G carriers (GT+GG) were significantly higher than those of the wild-type (TT). More importantly, the incidence rates of hypertension and hemoptysis (peripheral lung cancer) with TT+CT in rs3814637 and GT+GG in rs11568732 were significantly higher than those with CC and TT.

**Conclusions:** The plasma trough and peak concentrations varied significantly for both 8 and 12 mg of anlotinib. Single-nucleotide polymorphisms in CYP2C19 are significantly associated with hypertension, hemoptysis, and anlotinib peak concentrations. Polymorphisms in CYP450 may explain inter-individual differences in anlotinib-related adverse reactions.

## Introduction

Lung cancer is one of the most common cancers, killing approximately 1.8 million people worldwide each year (Sung et al., 2021). It is also one of the most common causes of cancer-related deaths in China and worldwide (Cao et al., 2021). In addition to traditional drugs, new cytotoxic drugs, molecular-targeted therapies, and immune checkpoint inhibitors have provided new approaches for cancer treatment (Wu et al., 2021). Among them, molecular-targeted therapies have high specificity for tumor cells and low toxicity, and are considered promising cancer treatments (Herbst et al., 2018). Tyrosine kinase (TK) is an important component of the intracellular signal transduction system, transmitting conditional information from the extracellular domain or cytoplasm to the nucleus. TK is dysregulated in many tumor cells. Therefore, tyrosine kinase inhibitors (TKIs) have emerged as an effective approach for molecular-targeted therapies (Krause and Van Etten, 2005; Ferguson and Gray, 2018).

Anlotinib, a small molecular multi-targeted TKI, can inhibit tumor angiogenesis and proliferation (Syed, 2018). *In vitro* studies have shown that anlotinib inhibits tumor cell growth by inhibiting platelet-derived growth factor receptor β (PDGFRβ), vascular endothelial growth factor receptor 2/3 (VEGFR2/3), and stem cell factor receptor (c-Kit) (Chi et al., 2018; Lin et al., 2018; Liu et al., 2018). *In vivo*, anlotinib displayed broad activity in human tumor xenograft models (Sun et al., 2016). Anlotinib has shown promise in several cancer clinical trials. Consequently, anlotinib has been approved in China as a third-line treatment for patients with advanced non-small cell lung cancer (NSCLC), and small cell lung cancer. Despite the numerous benefits of anlotinib treatment, there is an increasing number of cases of treatment failure due to drug resistance and toxicity. Hypertension, elevated thyroid-stimulating hormone (TSH) levels, hand-foot syndrome (HFS), hepatotoxicity, and hemoptysis are the most prevalent adverse reactions to anlotinib (Han et al., 2018a; Han et al., 2018b). With the widespread use of anlotinib in clinical practice, adverse reactions have become increasingly concerning.

Currently, research on advanced lung cancer focuses on identifying the biological predictors of efficacy and adverse events, which lead to the individualization of treatment. Systemic exposure or intracellular drug concentrations can influence drug efficacy and adverse reactions, resulting in interpatient variation (Decosterd et al., 2015). Furthermore, the level of drug exposure is significantly associated with the pharmacokinetic properties (absorption, distribution, and metabolism) of the drug. Therefore, inter-individual variations in pharmacokinetics may influence the efficacy and adverse reactions of anlotinib.

Growing evidence suggests that genetic polymorphisms in cytochrome P450 (CYP450) may significantly influence inter-individual differences in drug responses and disposition (Evans and McLeod, 2003). The activities of these drug-metabolizing enzymes determine how quickly the drug is metabolized and can therefore influence the occurrence of adverse reactions. CYP450 oxidizes approximately half of the widely prescribed medications as well as most oral small-molecule anticancer medicines, such as TKIs (Parra-Guillen et al., 2017). It has been shown that the adverse reactions of TKI correlate with drug concentrations and metabolic enzyme gene polymorphisms (Liao et al., 2020), such as CYP3A5*3 for sorafenib-related severe hepatic and renal damage (Guo et al., 2018), and CYP3A4-rs4646437 and CYP3A5-rs776746 for sunitinib-related hypertension (Garcia-Donas et al., 2011; Diekstra et al., 2015; Diekstra et al., 2017). Studies have shown that P450-mediated oxidation is a key factor that affects the oral bioavailability, exposure, half-life, and interspecific differences in anlotinib. A variety of CYP450 enzymes are involved in anlotinib metabolism *in vitro*, with CYP3A4 and CYP3A5 being the most readily metabolized enzymes (Zhong et al., 2018). Wei *et al.* investigated the effects of anlotinib on CYP1A2, CYP2C6, CYP2D1, CYP2D2, and CYP3A1/2 in animals and discovered that anlotinib strongly induces CYP2D1 and CYP3A1/2 (Sun et al., 2017). Although *in vitro* and *in vivo* experiments have confirmed the effect of CYP450 enzymes on anlotinib metabolism, the relationship between CYP450 gene polymorphisms, plasma concentrations and clinical adverse reactions of anlotinib in patients with lung cancer remains unclear.

Accordingly, for the first time, we propose that individual differences in plasma drug concentrations, and adverse reactions in patients with lung cancer treated with anlotinib may be correlated with CYP450 polymorphisms. To validate this hypothesis, we examined the plasma concentrations of anlotinib in subjects, analyzing single-nucleotide polymorphisms (SNPs) at high-frequency mutation sites in CYP450. In addition, we examined the correlations between polymorphisms of these genes, plasma concentrations, and adverse reactions.

## Materials and Methods

### Patient Eligibility

This single-center retrospective study was conducted in accordance with the Helsinki Declaration of the First Affiliated Hospital of Anhui Medical University, Hefei, China, between January 2020, and August 2021. The study protocol was reviewed and approved by the institutional ethics committee (No. Quick- PJ2019-14-15). Written informed consent was obtained from all patients. The inclusion criteria were as follows: *1*) patients aged 18–80 years *2*) gender was not limited; *3*) patients taking more than two courses of anlotinib, with trackable complete information about adverse reactions during treatment; and *4*) patients with clear pathological and imaging diagnoses of lung cancer, including non-small cell lung cancer and small cell lung cancer. The exclusion criteria were as follows: *1*) moderate-to-severe hepatic and renal insufficiency, *2*) allergy to anlotinib, *3*) missing basic or adverse reaction information, *4*) pregnant or lactating women, and *5*) poor compliance.

### Plasma Anlotinib Concentration Determination

Patients received a 2-week on/1-week off (2/1) schedule and a 12 or 8 mg once-daily dose for our study. As second-line treatment, anlotinib was combined with immune checkpoint inhibitors (carryilizumab and pabrolizumab). In addition, anlotinib alone was used as third-line treatment. None of the enrolled patients was taking drugs that affected the plasma levels of anlotinib (e.g., rifampicin, ketoconazole, and itraconazole). Peak concentration blood samples were collected at 8 a.m. on the day after the end of the course of treatment (day 15). Blood samples were collected 30 min before the start of the new course (day 22). The collected blood samples were centrifuged at 3,000 rpm for 5 min. The supernatant in the centrifuge tubes was collected and stored at −80°C until analysis, while the remaining samples were used for gene polymorphism analysis. An ACQUITY ultra-performance liquid chromatograph (UPLC, Waters, United States) combined with a Xevo TQ-S triple quadrupole mass spectrometer (Waters, United States) was used to determine the plasma concentrations of anlotinib. Gradient separation was performed on a PACQUITY UPLC BEH C18 column (1.7 µm 2.1 × 50 mm) (Waters, United States) at a temperature of 40°C and a flow rate of 0.5 ml/min. Data processing was performed using MassLynxV4.1 a data workstation (Waters, United States). A standard curve was established according to an internal reference to calculate the plasma concentrations of anlotinib. The results of methodological validation are presented in Supplementary Material S3.

### Genotype Identification

DNA was extracted from the blood samples of patients using a blood genomic DNA extraction kit (Tiangen Biotechnology, Beijing, China) and stored at −80°C until analysis. The quality of the extracted DNA was strictly controlled using Nanodrop 2000 (Thermo Scientific, United States) and capillary electrophoresis (Qiagen, Switzerland). In this study, 20 loci in five different CYP450 genes were selected for polymorphism detection, including rs2242480, rs35599367, rs4646437, rs3735451, and rs4646460 in CYP3A4; rs1419745, rs4646450, rs15524, and rs3800959 in CYP3A5; rs11568732, rs12248560, rs12769205, rs3814637, rs4244285, and rs4986893 in CYP2C19; rs2069526, rs2470890, rs4646425, and rs4646427 in CYP1A2; and rs9332113 in CYP2C9. Primers for all gene loci in patients were designed using Sequenom (Assay Design Suite V2.0) online software. MassARRAY Analyzer Compac mass spectrometry was used to detect gene locus information. TYPER software was used to analyze the results and obtain the genotyping data.

### Data Collection

All patients were followed-up in special clinics or wards between January 2020, and August 2021 to monitor anlotinib-induced adverse reactions. Adverse reactions were graded using the Common Terminology Criteria for Adverse Events version 5.0 (CTCAE 5.0). The results were recorded during an adverse reaction assessment.

### Statistical Analysis

All statistical analyses were performed using Statistical Products and Services Solutions, version 26 (SPSS 26.0). Descriptive statistics were used to summarize the demographic data and baseline characteristics of the patients receiving anlotinib. The mean and standard deviation were calculated for normally distributed data. Comparisons between groups for continuous variables were performed using an independent-sample *t*-test or one-way ANOVA. If the variance was not uniform, the Mann-Whitney *U* test was used. Chi-square and Fisher’s exact tests were used to compare differences in the Hardy-Weinberg balance test and adverse reactions among different genotypes. For all statistical analyses, a *p*-value < 0.05 was considered statistically significant.

## Results

### Patient Characteristics

In the current study, 139 patients were enrolled in the follow-up trials. The mean age of the study population was 62.97 ± 10.9 years. Of the patients, 87 (62.6%) were male and 52 (37.4%) were female. Only four patients (2.9%) had a family history of tumors. Twenty-four (17.3%) patients had a history of smoking. The detailed demographic and clinical characteristics of the patients are shown in Table 1.

**TABLE 1:** Patient characteristics.

Characteristics	No. of patients (%)
Mean age (years)	62.97 ± 10.9
Gender	
Male	87 (62.6%)
Female	52 (37.4%)
Cancer-related family history	
Yes	4 (2.9%)
No	135 (97.1%)
Smoking status	
Non-smoker	115 (82.7%)
Ever smoker	24 (17.3%)
Dosage	
8 mg	73 (52.5%)
12 mg	66 (47.5%)
ECOG score	
0	38 (27.3%)
1	85 (61.2%)
≥2	16 (11.5%)
Histology	
Adenocarcinoma	77 (55.4%)
Squamous cell carcinoma	27 (19.4%)
Small cell	21 (15.1%)
Others	14 (10.1%)
Clinical stage *n* (%)	
IIIB	19 (13.7%)
IV	120 (86.3%)
Treatment line	
<3rd line	47 (33.8%)
≥3rd line	92 (66.2%)
EGFR mutation	
Yes	17 (12.2%)
No	87 (62.6%)
Unknown	35 (25.2%)
Prior targeted therapy	
Yes	41 (29.5%)
No	98 (70.5%)
Prior thoracic radiotherapy	
Yes	39 (28.1%)
No	100 (71.9%)
Prior anti-angiogenesis treatment	
Yes	44 (31.7%)
No	95 (68.3%)
Surgical history	
Yes	101 (72.7%)
No	38 (27.3%)

### Plasma Anlotinib Concentration

**FIGURE 1:**
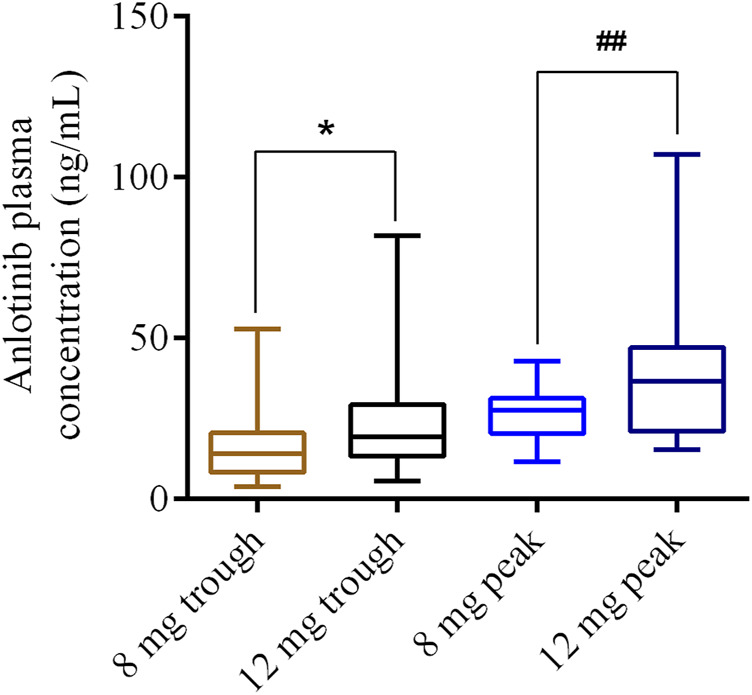
Distribution of anlotinib trough or peak concentrations for 8 and 12 mg dosages, respectively. *p < 0.05; ##*p* < 0.01.

A total of 73 of 139 patients with lung cancer received 8 mg anlotinib per day, and 66 patients received 12 mg anlotinib per day. The plasma concentrations of anlotinib are shown in Figure 1. The trough and peak concentrations of anlotinib (8 mg) were 17.59 ± 12.55 and 27.01 ± 7.97 ng/ml, ranging from 3.95 to 52.88 ng/ml and 11.53–42.8 ng/ml, respectively. In contrast, the trough and peak concentrations after 12 mg of anlotinib dosing were 23.51 ± 14.83 and 41.22 ± 27.15 ng/ml, ranging from 5.65 to 81.89 ng/ml and 18.01–107.18 ng/ml, respectively. There were significant differences between the trough and peak concentrations at a dosage of 8 versus 12 mg of anlotinib per day (*p* < 0.05).

### Genotype Frequencies

The genotype status of 20 CYP450 loci was determined in 139 patients. For the CYP3A5-rs15524 polymorphism, the frequencies of the A and G alleles were 69.42 and 30.58%, respectively. The frequencies of the G and A alleles for rs4646450 were 71.22 and 28.78%, respectively. The frequencies of the C and T alleles for rs1419745 were 28.42 and 71.58%, and the frequencies of the A and G alleles for rs3800959 were 82.37 and 17.63%, respectively. For the CYP3A4-rs2242480 polymorphism, the frequencies of the T and C alleles were 25.54 and 74.46%, respectively. The frequencies of the C and T alleles of rs43735451 were 31.29 and 68.71%, respectively. The frequencies of the A and G alleles for rs4646437 were 11.87 and 88.13%, respectively, and those of the A and G alleles for rs4646440 were 24.10 and 75.90%, respectively. For the CYP2C9-rs9332113 polymorphism, the frequencies of the G and C alleles were 65.83 and 34.17%, respectively. For the CYP2C19-rs12769205 polymorphism, the frequencies of the G and A alleles were 33.81 and 66.19%, respectively. The frequencies of the T and C alleles of rs12248560 were 1.44 and 98.56%, respectively. The frequencies of the T and C alleles of rs3814637 were 8.27 and 91.73%, respectively. The frequencies of the A and G alleles of rs4986893 were 5.76 and 94.24%, respectively. The frequencies of the G and T alleles for rs11568732 were 8.27 and 91.73%, and those of the A and G alleles for rs4244285 were 33.45 and 66.55%, respectively. For the CYP1A2-rs4646427 polymorphism, the frequencies of the C and T alleles were 6.47 and 93.53% respectively. The frequencies of the G and T alleles were 6.47 and 93.53% for rs2069526, respectively. The frequencies of the T and C alleles were 13.67 and 86.33% for rs2470890 and 93.53 and 6.47% for rs4646425, respectively. All alleles followed–y the Weinberg equilibrium, except for CYP340-rs35599367. Genotype and allele frequencies of the study population are shown in Table 2.

**TABLE 2:** Observed genotype and allele frequency of SNPs in the present study.

Gene	SNP-ID	Genotype	*n*	Identified Frequency %	Allele	Allele frequency %
CYP3A5	rs15524	AG	68	48.92	A	69.42
		AA	63	45.32	G	30.58
		GG	8	5.76	—	—
CYP3A5	rs4646450	GG	67	48.20	G	71.22
		AA	8	5.76	A	28.78
		AG	64	46.04	—	—
CYP3A5	rs1419745	CT	65	46.76	C	28.42
		TT	67	48.20	T	71.58
		CC	7	5.04	—	—
CYP3A5	rs3800959	AA	93	66.91	A	82.37
		AG	43	30.94	G	17.63
		GG	3	2.16	—	—
CYP3A4	rs35599367	GG	139	100.00	G	100.00
CYP3A4	rs2242480	TT	7	5.04	T	25.54
		CC	75	53.96	C	74.46
		CT	57	41.01	—	—
CYP3A4	rs3735451	CC	9	6.47	C	31.29
		TT	62	44.60	T	68.71
		CT	68	48.92	—	—
CYP3A4	rs4646437	GG	107	76.98	A	11.87
		AG	31	22.30	G	88.13
		AA	1	0.72	—	—
CYP3A4	rs4646440	AA	7	5.04	A	24.10
		AG	53	38.13	G	75.90
		GG	79	56.83	—	—
CYP2C9	rs9332113	CG	69	49.64	G	65.83
		GG	57	41.01	C	34.17
		CC	13	9.35	—	—
CYP2C19	rs12769205	GG	12	8.63	G	33.81
		AG	70	50.36	A	66.19
		AA	57	41.01	—	—
CYP2C19	rs12248560	CT	3	2.16	T	1.44
		CC	136	97.84	C	98.56
CYP2C19	rs3814637	CC	117	84.17	T	8.27
		TT	1	0.72	C	91.73
		CT	21	15.11	—	—
CYP2C19	rs4986893	GG	123	88.49	A	5.76
		AG	16	11.51	G	94.24
CYP2C19	rs11568732	GG	1	0.72	G	8.27
		GT	21	15.11	T	91.73
		TT	117	84.17	—	—
CYP2C19	rs4244285	GG	57	41.01	A	33.45
		AA	12	8.63	G	66.55
		AG	70	50.36	—	—
CYP1A2	rs4646427	CT	18	12.95	C	6.47
		TT	121	87.05	T	93.53
CYP1A2	rs2069526	GT	18	12.95	G	6.47
		TT	121	87.05	T	93.53
CYP1A2	rs2470890	CC	103	74.10	T	13.67
		TT	2	1.44	C	86.33
		CT	34	24.46	—	—
CYP1A2	rs4646425	CC	121	87.05	C	93.53
		CT	18	12.95	T	6.47

SNPs, single nucleotide polymorphisms; *n*, the numbers of patients.

### Influence of Different Genotypes on Anlotinib Peak Plasma Concentration

Correlation analysis between different genotypes and the peak plasma concentrations of anlotinib showed that CYP2C19-rs3814637 and -rs11568732 were significantly associated with peak plasma concentrations. For CYP2C19-rs3814637, the peak plasma concentrations of the mutant allele T carriers (TT+CT) were significantly higher than those of the wildtypes (CC) (*p* = 0.035). For CYP2C19-rs11568732, the peak plasma concentrations of mutant allele G carriers (GT+GG) were significantly higher than those of the wild-type (TT) (*p* = 0.035), as shown in Table 3. The differences between the plasma concentrations of the other genotypes and that of anlotinib were not statistically significant.

**TABLE 3:** Correlation between different genotypes and anlotinib plasma peak concentration.

Gene	SNP_ID	Genotype	*n*	Mean ± SD (ng/ml)	*p* value
CYP2C19	rs3814637	CC	35	30.29 ± 16.42	—
		CT+TT	5	49.52 ± 30.32	0.035^a^
	rs11568732	TT	35	30.29 ± 16.42	—
		GT+GG	5	49.52 ± 30.32	0.035^a^

a Significant at *p* < 0.05.

### Effect of Genetic Polymorphisms on Adverse Reactions of Anlotinib

After analyzing the correlation between different genotypes and adverse reactions of anlotinib, for all 20 of the SNPs, only the mutations of CYP2C19-rs3814637 and -rs11568732 genotypes were significantly associated with the occurrence of hypertension and hemoptysis (peripheral lung cancer) in the study population.

The incidence rate of hypertension in mutant allele T carriers (CT + TT) of rs3814637 was significantly higher than that in the wildtype (CC) (*p* = 0.034, odds ratio (OR) = 0.316, 95% confidence interval (CI): 0.12–0.835). The incidence rate of hypertension in mutated allele G carriers (GT + GG) of rs11568732 was significantly higher than that in the wild type (TT) (*p* = 0.034, OR = 0.316, 95% CI: 0.12–0.835).

The incidence rate of hemoptysis (peripheral lung cancer) in mutated allele G carriers (GT + GG) of rs11568732 was significantly higher than that in the wild type (TT) (*p* = 0.043, OR = 0.13, 95% CI: 0.02–0.845). The incidence rate of hemoptysis (peripheral lung cancer) in mutant allele T carriers (CT + TT) of rs3814637 was significantly higher than that in the wild type (CC) (*p* = 0.043, OR = 0.13, 95% CI: 0.02–0.845). There was no significant difference between rs11568732 and rs3814637 genotypes in the incidence of hemoptysis between central lung cancer (*p* > 0.05). Table 4 presents the results.

**TABLE 4:** Correlation between genetic polymorphisms and adverse reactions.

Adverse reactions	Gene	SNP-ID	Genotype	Abnormal group (*n*)	Normal group (*n*)	*p* value	OR	95%CI
Hypertension	CYP2C19	rs3814637	CC	21 (0.179)	96 (0.821)	—	—	—
			TT+CT	9 (0.409)	13 (0.591)	0.034^a^	0.316	0.12–0.84
		rs11568732	TT	9 (0.409)	13 (0.591)	—	—	—
			GT+GG	21 (0.179)	96 (0.821)	0.034^a^	0.316	0.12–0.84
	CYP3A4	rs2242480	CC	17 (0.227)	58 (0.773)	—	—	—
			CT+TT	13 (0.203)	51 (0.797)	0.737	0.87	0.39–1.96
		rs3735451	TT	13 (0.206)	49 (0.794)	—	—	—
			CC+CT	17 (0.224)	60 (0.776)	0.874	0.936	0.42–2.12
		rs4646437	GG	22 (0.206)	85 (0.794)	—	—	—
			AG+AA	8 (0.250)	24 (0.750)	0.592	0.776	0.31–1.96
		rs4646440	GG	18 (0.228)	61 (0.772)	—	—	—
			AA+AG	12 (0.200)	48 (0.800)	0.693	0.847	0.37–1.93
	CYP3A5	rs1419745	TT	14 (0.209)	53 (0.791)	—	—	—
			CC+CT	16 (0.222)	56 (0.778)	0.849	0.925	0.41–2.08
		rs15524	AA	13 (0.206)	50 (0.794)	—	—	—
			GG+AG	17 (0.224)	59 (0.776)	0.805	1.108	0.49–2.50
		rs3800959	AA	22 (0.237)	71 (0.763)	—	—	—
			AG+GG	8 (0.174)	38 (0.826)	0.398	0.679	0.28–1.67
		rs4646450	GG	14 (0.209)	53 (0.791)	—	—	—
			AG+AA	16 (0.222)	56 (0.778)	0.849	1.082	0.48–2.43
Hemoptysis	CYP2C19	rs11568732	TT	2 (0.025)	77 (0.975)	—	—	—
(Peripheral lung cancer)			GT+GG	3 (0.167)	15 (0.833)	0.043^a^	0.13	0.02–0.85
		rs3814637	CC	2 (0.025)	77 (0.975)	—	—	—
			CT+TT	3 (0.167)	15 (0.833)	0.043^a^	0.13	0.02–0.85
	CYP3A4	rs2242480	CC	2 (0.041)	47 (0.959)	—	—	—
			CT+TT	3 (0.063)	45 (0.938)	0.981	0.638	0.10–4.00
		rs3735451	TT	2 (0.050)	38 (0.950)	—	—	—
			CC+CT	3 (0.053)	54 (0.947)	1.000	0.947	0.15–5.95
		rs4646437	GG	4 (0.056)	68 (0.944)	—	—	—
			AG+AA	1 (0.040)	24 (0.960)	1.000	1.412	0.15–13.26
		rs4646440	GG	2 (0.038)	50 (0.962)	—	—	—
			AA+AG	3 (0.067)	42 (0.933)	0.868	0.560	0.09–3.51
	CYP3A5	rs1419745	TT	2 (0.048)	40 (0.952)	—	—	—
			CC+CT	3 (0.055)	52 (0.945)	1.000	0.867	0.14–5.44
		rs15524	AA	2 (0.049)	39 (0.951)	—	—	—
			GG+AG	3 (0.054)	53 (0.946)	1.000	0.906	0.14–5.68
		rs3800959	AA	5 (0.070)	66 (0.930)	—	—	—
			AG+GG	0 (0.000)	26 (1.000)	0.384	0.93	0.87–0.99
		rs4646450	GG	2 (0.048)	40 (0.952)	—	—	—
			AG+AA	3 (0.055)	52 (0.945)	1.000	0.867	0.14–5.44
Hemoptysis	CYP2C19	rs11568732	TT	6 (0.158)	32 (0.842)	—	—	—
(central lung cancer)			GT+GG	1 (0.250)	3 (0.750)	0.532	0.563	0.05–6.36
		rs3814637	CC	6 (0.158)	32 (0.842)	—	—	—
			CT+TT	1 (0.250)	3 (0.750)	0.532	0.563	0.05–6.36
	CYP3A4	rs2242480	CC	3 (0.115)	23 (0.885)	—	—	—
			CT+TT	4 (0.250)	12 (0.750)	0.447	0.391	0.08–2.04
		rs3735451	TT	3 (0.136)	19 (0.864)	—	—	—
			CC+CT	4 (0.200)	16 (0.800)	0.89	0.632	0.12–3.25
		rs4646437	GG	6 (0.171)	29 (0.829)	—	—	—
			AG+AA	1 (0.143)	6 (0.857)	1.000	1.241	0.13–12.29
		rs4646440	GG	3 (0.111)	24 (0.889)	—	—	—
			AA+AG	4 (0.267)	11 (0.733)	0.388	0.344	0.07–1.81
	CYP3A5	rs1419745	TT	3 (0.120)	22 (0.880)	—	—	—
			CC+CT	4 (0.235)	13 (0.765)	0.574	0.443	0.09–2.30
		rs15524	AA	3 (0.136)	19 (0.864)	—	—	—
			GG+AG	4 (0.200)	16 (0.800)	0.890	0.632	0.12–3.25
		rs3800959	AA	4 (0.182)	18 (0.818)	—	—	—
			AG+GG	3 (0.150)	17 (0.850)	1.000	1.259	0.25–6.47
		rs4646450	GG	3 (0.120)	22 (0.880)	—	—	—
			AG+AA	4 (0.235)	13 (0.765)	0.574	0.443	0.09–2.30

a Significant at *p* < 0.05. OR, odd ratio; 95%CI, 95%confidence interval.

There were no significant differences in the incidence of proteinuria, hepatotoxicity, or other adverse reactions among the 20 SNP genotypes (*p* > 0.05).

## Discussion

Anlotinib is a multitarget drug that has been developed and marketed independently in China. A phase I clinical trial confirmed that anlotinib showed controlled toxicity at a dose of 12 mg once daily in a 2-week on/1-week off schedule (2/1) (Sun et al., 2016). However, adverse reactions to anlotinib in clinical practice often leads to dose reductions, or treatment discontinuation. In the present study, the most common adverse reactions associated with anlotinib were hypertension, hemoptysis, elevated TSH levels, hepatotoxicity, hypertriglyceridemia, proteinuria, dyspepsia, and HFS. Notably, hypertension was the most common adverse reaction during anlotinib treatment, which is consistent with the results of a phase II trial (Han et al., 2018b). In this study, plasma anlotinib concentrations showed large individual variations. In addition, 11 patients developed a grade 3 or higher incidence of hypertension during treatment, resulting in dose reductions in five patients and drug changes in six patients. This dramatic change can be explained not only by drug interactions and acquired factors but also by genetic factors, such as CYP450 gene polymorphisms. As CYP450 is regulated by genes, once the gene is mutated, CYP450 is regulated and synthesized, and drug metabolism *via* the enzyme is subsequently altered. Therefore, polymorphisms in CYP450 are the basis of race and individual differences in drug metabolism. In this study, we used a candidate gene approach to investigate the correlation between polymorphisms in genes encoding drug-metabolizing enzymes and anlotinib toxicity.

In humans, CYP3A4/5 has the largest impact on drug biotransformation, followed by CYP2D6, CYP2C6, CYP1A2, and other enzymes (Zanger and Schwab, 2013). Similarly, TKIs are metabolized by CYP450 enzymes, which affects drug concentrations. Imatinib is a cytochrome of CYP3A4 and CYP2C8 substrates that markedly increases plasma CYP3A4 substrate concentrations and reduces hepatic CYP3A4 activity in humans (Filppula et al., 2012). According to previous *in vitro* investigations, erlotinib is largely metabolized by CYP3A4, whereas CYP1A2 makes a minor contribution (Endo-Tsukude et al., 2018). The major metabolic enzymes of anlotinib are similar to those of imatinib and erlotinib. CYP3A4 and CYP3A5 play important roles in the metabolism of anlotinib.

It is known to all that the genetic mutations of metabolic enzymes do not necessarily lead to corresponding changes in blood exposure to substrate drugs. Currently, there is no evidence of the influence of CYP3A4/5 gene mutations on blood exposure to anlotinib. Anlotinib metabolism is regulated by multiple enzymes. In addition, as mentioned in the previous paragraph, even if CYP3A4/5 gene mutation alters its enzyme activity, this single degree of enzyme activity change does not necessarily affect the pharmacokinetic characteristics of anlotinib (Zhong et al., 2018). This process involves many complex mechanisms. For example, as the largest part (82%) of the intestinal CYP450 family, intestinal CYP3A is inhibited by anlotinib during intestinal absorption (the first site of drug exposure in the body’s metabolic system) and probably results in increased systemic exposure to anlotinib (Kaminsky and Zhang, 2003; Paine et al., 2006). There are many other factors.

This study provides evidence that anlotinib blood exposure is clinically associated not with CYP3A4 and CYP3A5 mutations but with CYP2C19 mutations. This is an interesting phenomenon, and further mechanistic evidence is required.

Previous *in vivo* studies have demonstrated that efficacy and adverse reactions are closely linked to genetic factors. However, few drug-metabolizing enzyme gene polymorphisms have been used as predictive factors of TKI toxicity (Scott, 2011; Patel, 2016). In this study, we found that the CYP2C19-rs3814637 and -rs11568732 genotypes were significantly associated with the occurrence of hypertension and hemoptysis. Studies have shown that CYP2C19 genotype can guide antiplatelet therapy (Martin et al., 2020). In addition, polymorphisms in the CYP2C19 gene have been reported to be positively associated with cardiovascular diseases, such as coronary artery disease and atherosclerosis (Ercan et al., 2008; Yang et al., 2010). Further studies showed that CYP2C19-rs11568732 was significantly associated with bleeding in patients with ST-segment elevation myocardial infarction who underwent primary percutaneous coronary intervention and treatment with clopidogrel (Novkovic et al., 2018). This finding is consistent with the results of our study, which showed that CYP2C19-rs11568732 was significantly associated with the occurrence of hemoptysis. Additionally, studies have shown that the CYP2C19*3 defective allele may contribute to a reduced risk of developing essential hypertension (Shin et al., 2012). However, few studies have investigated the correlation between CYP2C19-rs3814637 and -rs11568732 polymorphisms and hypertension. In this study, no association was observed between the different genotypes of other CYP450 loci and adverse reactions to anlotinib.

The epidermal growth factor receptor (EGFR) is a ligand receptor for epidermal growth factor (EGF). When EGF binds to EGFR, the signaling pathway is activated, leading to cell proliferation and differentiation. EGFR overexpression due to mutations or structural changes promotes carcinogenesis, invasion, and metastasis. Studies have shown that common mutations are associated with sensitivity to EGFR tyrosine kinase inhibitors (TKI) (Lynch et al., 2004; Paez et al., 2004). Anlotinib is a small-molecule, multi-target TKI. Currently, there is no evidence for an association between anlotinib and EGFR mutations. In this study, 17 patients were found to have EGFR mutations; however, no correlation was found between EGFR mutations and anlotinib-induced adverse reactions.

Based on this study, it is reasonable to speculate that inter-individual differences in anlotinib-related adverse reactions may be explained by CYP450 gene polymorphisms or different exposures, caused by CYP450 gene polymorphisms. Thus, the clinical application of anlotinib should be based on the genotyping of CYP450 in lung cancer, particularly for rs3814637 and rs11568732 of CYP2C19. This strategy may reduce the incidence of anlotinib-related adverse reactions. However, the underlying mechanisms by which CYP450 gene polymorphisms influence the variation in anlotinib-related adverse reactions and toxicity between individuals remain unclear, and could be a direction for further research.

Nevertheless, our study has a few limitations. First, it was a single-center prospective study with limited patient enrolment. Second, owing to the short follow-up time of the patients, there was no statistical information about progression-free survival and overall survival. Third, the anlotinib concentration may be influenced by other factors (e.g., coffee and drugs), which was not studied separately.

## Conclusion

In summary, some CYP450 genotypes are significantly associated with adverse reactions to anlotinib in clinical practice, including hypertension and hemoptysis. Our results show that these genetic variants in CYP450 can explain inter-individual differences in anlotinib adverse reactions. Therefore, identifying CYP450 gene polymorphisms, particularly CYP2C19-rs3814637 and -rs11568732, before anlotinib treatment might be a potential personalized treatment approach.

## Data Availability

The original contributions presented in the study are included in the article/Supplementary Materials, further inquiries can be directed to the corresponding author.
