# Supplementary material for: Genetic Polymorphisms in CYP2C19 Cause Changes in Plasma Levels and Adverse Reactions to Anlotinib in Chinese Patients With Lung Cancer
Source: Front Pharmacol. 2022 Jun 22;13:918219. doi: 10.3389/fphar.2022.918219 (PMC9257029; doi:10.3389/fphar.2022.918219)
Supplement: Supplementary file 1 [file DataSheet1.PDF]

Supplementary 1

The correlation between the gene polymorphisms of CYP3A4/3A5 and Hypertension of anlotinib

Adverse reactions	Gene	SNP-ID	Genotype	Abnormal group (n)	Normal group (n)	*P* value	OR	95%CI
Hypertension	CYP3A4	rs2242480	CC	17(0.227)	58(0.773)			
			TT+CT	13(0.203)	51(0.797)	0.737	0.87	0.385-1.963
		rs3735451	TT	13(0.206)	49(0.794)			
			GT+GG	17(0.224)	60(0.776)	0.874	0.936	0.415-2.115
		rs4646437	GG	22(0.206)	85(0.794)			
			AG+AA	8(0.250)	24(0.750)	0.592	0.776	0.307-1.963
		rs4646440	GG	18(0.228)	61(0.772)			
			AA+AG	12(0.200)	48(0.800)	0.693	0.847	0.372-1.928
	CYP3A5	rs1419745	TT	14(0.209)	53(0.791)			
			CC+CT	16(0.222)	56(0.778)	0.849	0.925	0.411-2.078
		rs15524	AA	13(0.206)	50(0.794)			
			GG+AG	17(0.224)	59(0.776)	0.805	1.108	0.491-2.502
		rs3800959	AA	22(0.237)	71(0.763)			
			AG+GG	8(0.174)	38(0.826)	0.398	0.679	0.276-1.671
		rs4646450	GG	14(0.209)	53(0.791)			
			AG+AA	16(0.222)	56(0.778)	0.849	1.082	0.481-2.431

Abbreviations: OR, odd ratio; 95%CI, 95%confidence interval.

Supplementary 2

The correlation between the gene polymorphisms of CYP3A4 and CYP3A5 and hemoptysis (Peripheral lung cancer) of anlotinib

adverse reactions	Gene	SNP-ID	Genotype	Abnormal group (n)	Normal group (n)	*P* value	OR	95%CI
Hemoptysis (Peripheral lung cancer)	CYP3A4	rs2242480	CC	2(0.041)	47(0.959)			
			TT+CT	3(0.063)	45(0.938)	0.981	0.638	0.102-4.000
		rs3735451	TT	2(0.050)	38(0.950)			
			GT+GG	3(0.053)	54(0.947)	1.000	0.947	0.151-5.945
		rs4646437	GG	4(0.056)	68(0.944)			
			AG+AA	1(0.040)	24(0.960)	1.000	1.412	0.150-13.26
		rs4646440	GG	2(0.038)	50(0.962)			
			AA+AG	3(0.067)	42(0.933)	0.868	0.560	0.089-3.511
	CYP3A5	rs1419745	TT	2(0.048)	40(0.952)			
			CC+CT	3(0.055)	52(0.945)	1.000	0.867	0.138-5.436
		rs15524	AA	2(0.049)	39(0.951)			
			GG+AG	3(0.054)	53(0.946)	1.000	0.906	0.144-5.684
		rs3800959	AA	5(0.070)	66(0.930)			
			AG+GG	0(0.000)	26(1.000)	0.384	0.93	0.872-0.991
		rs4646450	GG	2(0.048)	40(0.952)			
			AG+AA	3(0.055)	52(0.945)	1.000	0.867	0.138-5.436

Abbreviations: OR, odd ratio; 95%CI, 95%confidence interval.

Supplementary 3

Methodological validation for plasma concentration assay of anlotinib.

1.1 The chromatographic conditions

Chromatographic column: ACQUITY UPLC BEH C18(1.7μm 2.1×50mm), Waters (USA)

Protect column: THE EVO-C18 UHPLC (Sub-2 μm and Core-shell Columns with 2.5 mm Internal breathing Sections), phenomenex

Mobile phase: A:0.01% ammonia water B: methanol

Flow rate: 0.5 mL/min

Column temperature: 40 ℃

Sample quantity: 0.5μL

The gradient:

time（min）	Mobile phase A（%）	Mobile phase B（%）
0	60	40
0.2	60	40
2.0	10	90
2.5	10	90
2.6	60	40
3.0	60	40

1.2 Mass spectrometry conditions

Ion source: ESI source

Detection mode: positive ion detection

Scanning mode: multiple reactive ion monitoring (MRM)

Quantitative ion: M/Z408.26 →339.19; 【D5】-deuterated anlotinib (internal standard)

M/Z413.31 →344.23

Parameters	Setting
Capillary（kv）	3kv
Cone（v）	30v
Source（I）	150I
desolvation tem（I）	450I
Desolvation（L/hr）	1000 L/hr

Cone（L/hr）	150 L/hr

1.3 Selective examination

By examining the analysis of 6 different individual blank plasma treatment feeds, we found that the retention time of both anlotinib and the internal standard [D5]-deuterated anlotinib was 1.77 min, with good chromatogram peak shape, smooth baseline, no spurious peak interference in the plasma feed, and good specificity. The ratio of the peak area of blank blood sample to the peak area of Lower Limit of quantitation (LLOQ): 292/16765*100%= 1.7% (≤20%); the ratio of the peak area of blank blood sample to the peak area of internal standard: 51.456/469962.063*100%= 0.01% (≤5%); the results showed that the endogenous substances of anlotinib, [D5]-deuterated anlotinib and plasma were completely separated. The endogenous substances in the blank plasma do not interfere with the determination of anlotinib and its internal standard.

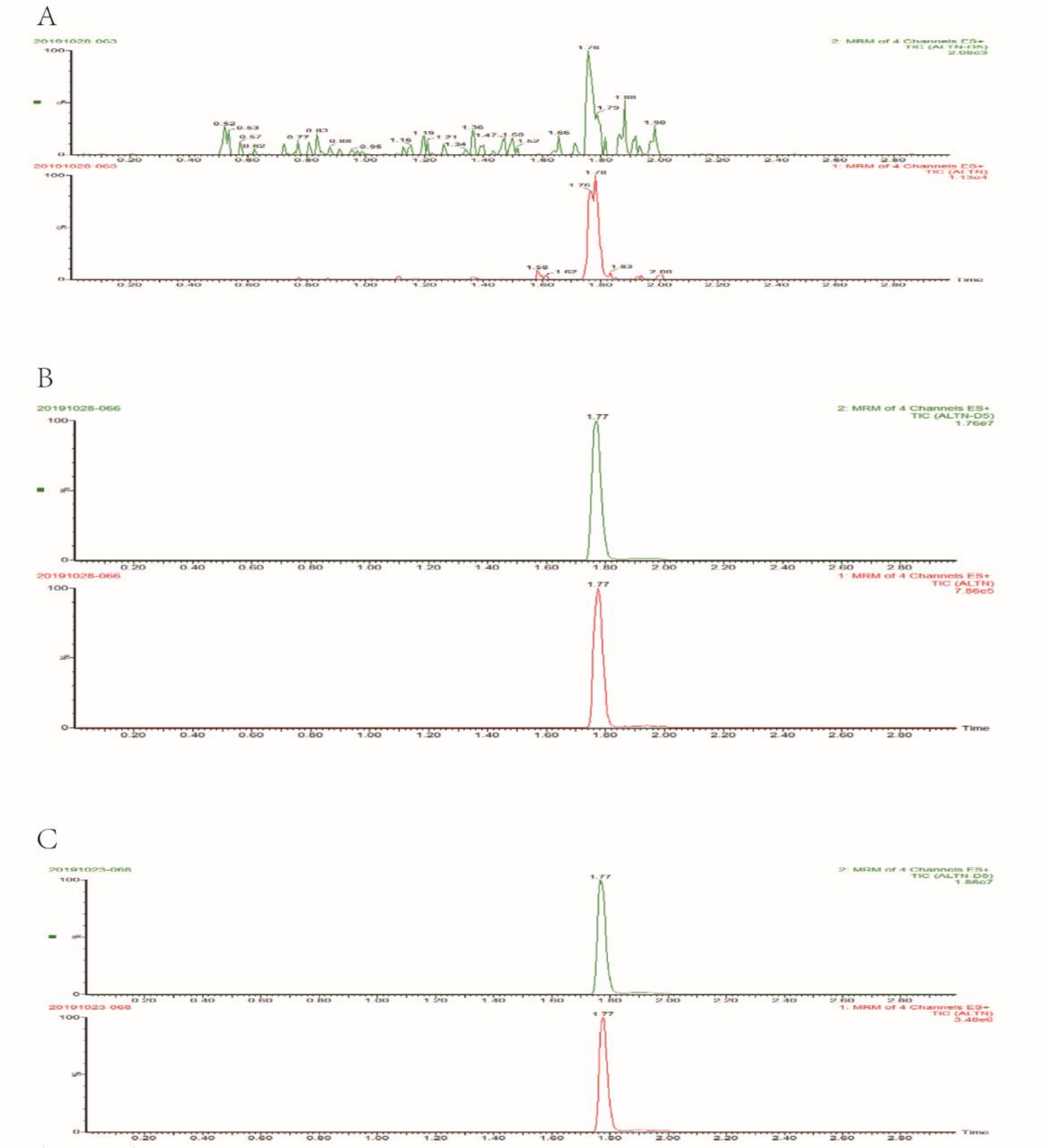

Figure1. Selective experimental chromatogram

(A) blank plasma. (B) blank plasma + anlotinib +【D5】-deuterated anlotinib
(C) Patient plasma samples +【D5】-deuterated anlotinib

1.4 Precision and Accuracy

Plasma samples containing four mass concentrations (5, 10, 100, and 200 ng/mL) were processed for injection. Intraday precision and accuracy were 2.46%-4.96%(≤15%), 9.84%(LLOQ, ≤20%), 101.6±0.05%-102.8±0.03% (≤15%), and 104.4±0.10% (LLOQ, ≤20%). Similarly, the inter-day precision and accuracy were 0.46-2.38% (≤15%), 2.59% (LLOQ, ≤20%), 98.86±0.05%-103.17±0.03% (≤15%), and 102.52±0.03% (LLOQ, ≤20%). The results met the standard. It shows that the precision and accuracy of the method are good.

1.5 Matrix effects and Extraction Recovery rate

Through the processing of low-concentration and high-concentration quality control samples, the matrix effect results showed that the RSD were 7.96 % (≤15%) and 14.98 % (≤15%). The matrix effect meets the standard. By injecting the low, medium and high concentration quality control samples with different treatments, the extraction recovery results showed that the RSD of the medium and high concentration samples were 1.64 % (≤15%) and 1.7% (≤15%), and the RSD of the low concentration sample was 1.02% (≤20%). The Extraction Recovery rate meets the standard.

1.6 Stability

The results showed that the relative errors in the stability of the stock solutions were all ≤15%. The accuracy of the stability of the biological samples were all ≤15%. Similarly, the accuracy of the post-treatment sample stability was ≤15%. It shows that anlotinib have good stability and can be used for the concentration assay.

Supplementary 4

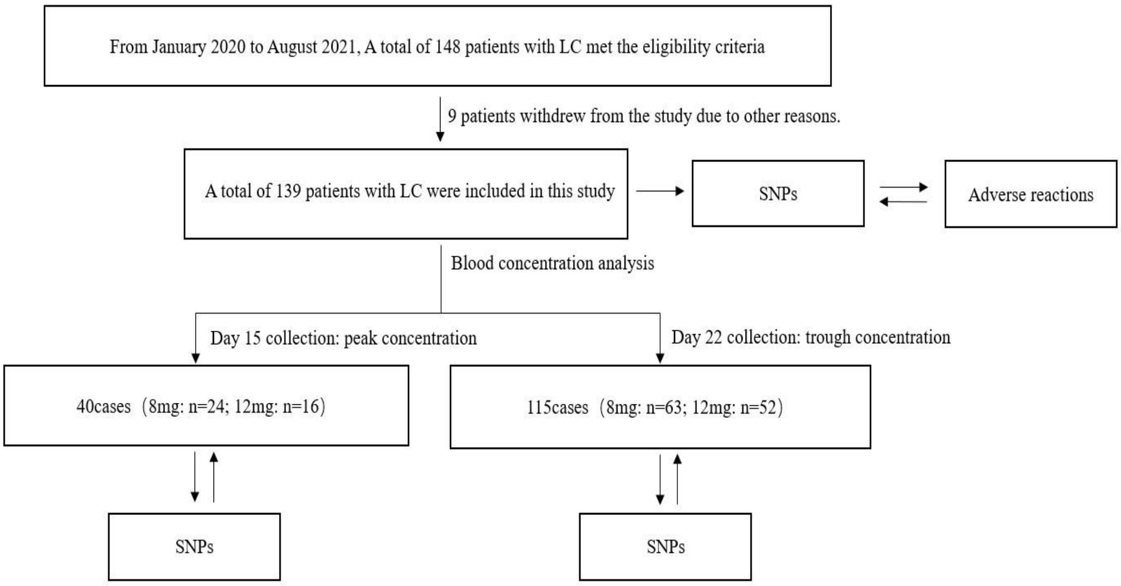

Fig 2 the flow chart of study

Supplementary 5

The correlation between EGFR mutations and anlotinib-induced adverse reactions

adverse reactions	States	Abnormal group (n)	Normal group (n)	*P* value	OR	95%CI
Hypertension	EGFR mutations	17	6	0.278	0.175	1.366
	others	122	24			
Hemoptysis	EGFR mutations	17	2	0.893	0.124	3.027
	others	122	10			

Abbreviations: OR, odd ratio; 95%CI, 95%confidence interval; others, EGFR is mutation free and unknown.

Supplementary 6

Hardy-Weinberg equilibrium test for enrolled patients

Gene	SNPs	HWE *P*-value
CYP2C19	rs11568732	0.95672
	rs4986893	0.47153
	rs4244285	0.16219
	rs12248560	0.93167
	rs12769205	0.14019
	rs3814637	0.95672
CYP1A2	rs4646425	0.41438
	rs2470890	0.66778
	rs4646427	0.41438
	rs2069526	0.41438
CYP2C9	rs9332113	0.22297
CYP3A4	rs4646437	0.43702
	rs4646440	0.61857
	rs35599367	<0.05
	rs3735451	0.10244
	rs2242480	0.35668
CYP3A5	rs4646450	0.14625
	rs1419745	0.07813
	rs3800959	0.44121
	rs15524	0.06975

Abbreviations: HWE, Hardy-Weinberg equilibrium; SNPs, single nucleotide polymorphisms
